# Real-Time Detection of Phenylacetaldehyde in Wine: Application of a Microwave Sensor Based on Molecularly Imprinted Silica

**DOI:** 10.3390/molecules27051492

**Published:** 2022-02-23

**Authors:** Jérôme Rossignol, Philippe Cayot, Didier Stuerga, Régis D. Gougeon, Elias Bou-Maroun

**Affiliations:** 1 Laboratoire Interdisciplinaire Carnot de Bourgogne, CNRS UMR 6303, Departement Interface, GERM, University Bourgogne Franche-Comté, 21078 Dijon, France; jerome.rossignol@u-bourgogne.fr (J.R.); didier.stuerga@u-bourgogne.fr (D.S.); 2 UMR Procédés Alimentaires et Microbiologiques, PAM UMR A 02.102, University Bourgogne Franche-Comté, AgroSup Dijon, 21000 Dijon, France; philippe.cayot@agrosupdijon.fr (P.C.); regis.gougeon@u-bourgogne.fr (R.D.G.)

**Keywords:** molecularly imprinted silica, microwave sensor, wine oxidation, phenylacetaldehyde, fast analysis

## Abstract

Molecularly imprinted sol–gel silica (MIS) coupled to a microwave sensor was designed and used to detect phenylacetaldehyde (PAA), a chemical tracer of wine oxidation. The developed method is fast, cheap and could replace the classical chromatographic methods, which require a tedious sample preparation and are expensive. To reach our objective, five MIS and their control non-imprinted silica (NIS) were synthesized and their extraction capacity toward PAA was studied in hydro alcoholic medium. The selected polymers, based on this first step, were subjected to a selectivity study in the presence of PAA and three other competing molecules. The best polymer was integrated in a microwave sensor and was used to assess PAA in red wine. The developed sensor was able to detect PAA at the µg·L^−1^ level, which is below the off-flavour threshold.

## 1. Introduction

Off-flavours, related to wine oxidation, generate wine rejection and cause a significant economic loss in wine production. Aldehydes are a known family of aroma compounds associated with wine oxidation. Phenylacetaldehyde (PAA) can be considered among chemical indicators of the oxidation level of a given wine [1]. Its aroma threshold varies between 1 and 25 µg⋅L^−1^ depending on the matrix. It is important to have analytical tools able to detect PAA below the sensory threshold of this aroma compound. Early detection of this fault marker in wine, at concentrations under the aroma threshold, is required for an efficient anticipation of proper enological practices to protect wine from oxidation.

The most used technics to follow PAA in wine are gas chromatography coupled to mass spectrometry detection. These targeted technics are highly efficient but relatively expensive, require a tedious sample preparation step and need high technical experience for the lab staff. A rapid, cheap and real-time analytical tool is essential to assess the level of wine oxidation.

Several sensors were developed to detect phenylacetaldehyde. They can be classified into two categories: biosensors [2,3] or chemical sensors [4,5]. Biosensors use a biomolecule as sensitive material; they are highly selective but not very stable in extreme conditions of pH or temperature. Chemical sensors are generally stable but lack selectivity in complex matrices such as wine and food. To the best of our knowledge, no chemical sensor or biosensor was developed to detect PAA in wine.

Molecularly imprinted materials (MIM) are one of the most specific and selective materials used in analytical chemistry. They are biomimetic synthetic materials able to mimic the specific interactions between antigens and antibodies, hormones and receptors or substrates and enzymes. MIM have the big advantage of being stable in extreme conditions of pH and temperature in comparison with biological materials [6]. MIM have a low cost of production. They are used in this study as sensitive materials to interact specifically with PAA. The most used MIM are acrylate-based polymers. We already demonstrated that sol–gel molecularly imprinted polymers were more specific than their equivalent acrylate-based polymers [7]. For this reason, only molecularly imprinted silica was prepared in this study.

Microwave sensors are based on the dielectric evolution of a sensitive material after interaction with a chemical target. Microwave sensing technology is relatively recent compared with other transduction methods such as electrochemistry, piezoelectric, thermocouple and optical transduction. Microwave sensors have found application in several fields such as medicine [8], environment [9], industry [10], food and agriculture [11].

The use of microwave sensing in a broad range of frequency (10 Mhz to 20 GHz) provides a rich information spectrum, allowing easy quantification of the target molecule in complex matrices such as wine [12].

In a previous work, we demonstrated the feasibility of such a MIS sensor to detect a fungicide in wine model solution down to 0.33 ng⋅L^−1^ [13]. We present in this work the strategy used to develop a microwave sensor, having molecularly imprinted silica (MIS) as a sensitive material, able to detect PAA in wine below 1 µg⋅L^−1^ without any sample preparation.

## 2. Results and Discussion

To select a molecularly imprinted silica (MIS) in order to integrate it in a microwave sensor, the following strategy was used: (1) Synthesis of five different MIS and their corresponding controls, non-imprinted silica (NIS). (2) Batch extraction studies of PAA in hydro alcoholic medium by the five MIS and NIS in order to assess the sorption ability of the polymers. (3) Batch selectivity studies in hydro alcoholic medium where the MIS and NIS were contacted with PAA and other competing molecules.

### 2.1. Synthesis of Molecularly Imprinted Silica

The synthesized polymers are presented in (Table 1). The sol–gel process was used to prepare the polymers. The most important parameters influencing the synthesis were varied: the functional monomer, the pH and the relative ratio of water/ethanol. The choice of the functional monomer is crucial in the MIS synthesis. It must present chemicals complementarity with the phenylacetaldehyde (PAA) template. [3-(Phenylamino)propyl]trimethoxysilane (PATMS) was chosen because it allows π–π interaction and hydrogen bond interaction with PAA. Phenyltrimethoxysilane (PTMS) allows only π–π interaction with PAA. Two pH conditions were considered. Under acid conditions, the hydrolysis kinetic of the sol–gel process is fast and the condensation kinetic is slow. In this case, linear polymers are produced. Under alkali conditions, condensation is faster than hydrolysis, and a highly condensed polymer is favored, leading to an agglomerate of fine particles [14].

MIS1 was eliminated because polymerization did not occur. This could be due to the high solubility of the polymer in this medium, which prevents it from polymerization. MIS2 was eliminated because a parasitic reaction occurred between the amine group of (3-aminopropyl)trimethoxysilane (APTMS) and the aldehyde function of phenylacetaldehyde. MIS3-5 were kept for the second step. Their sorption capacity toward PAA was studied in hydro alcoholic medium.

### 2.2. Batch Extraction Studies of PAA in Hydro Alcoholic Medium

**Figure 1:**
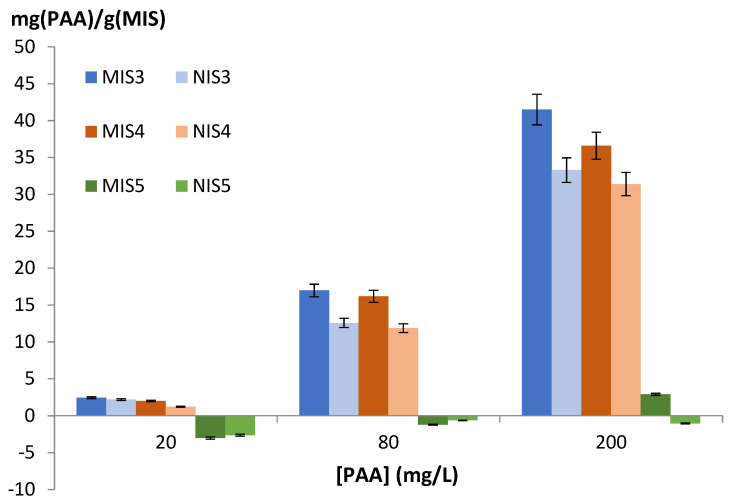
Adsorbed PAA/g of polymer vs. initial concentration in water/EtOH (90/10; *v*/*v*). Error bars represent standard deviation. MIS are the imprinted polymers, NIS the non-imprinted polymers.

A batch extraction of phenylacetaldehyde by all the polymers was carried out in hydro alcoholic medium. Results of the batch extraction are shown in Figure 1.

The quantity of adsorbed phenylacetaldehyde is expressed in function of the initial concentration. Polymer 5 showed low or no adsorption of phenylacetaldehyde, whatever the initial concentration. For this reason, it was discarded for the selectivity tests. Sorption by polymers 3 and 4 increased with the initial concentration of PAA reaching 41.5 mg/g for polymer 3. Polymers 3 and 4 were kept for the selectivity study. The non-imprinted polymer (NIS) with the formulations n°3 and n°4 was able to capture the AAP, but less efficiently compared with the imprinted polymers (MIS).

The MIS has chemical and steric complementarity with the target molecule. The NIS has only chemical complementarity. MIS and NIS have the same chemical structure: they contain both of the functional monomers. The latter is responsible for the chemical interaction with the target molecule. As the difference between MIS and NIS is small, this means that the chemical interaction is more important than the steric interaction.

### 2.3. Batch Selectivity Studies in Hydro Alcoholic Medium

**Figure 2:**
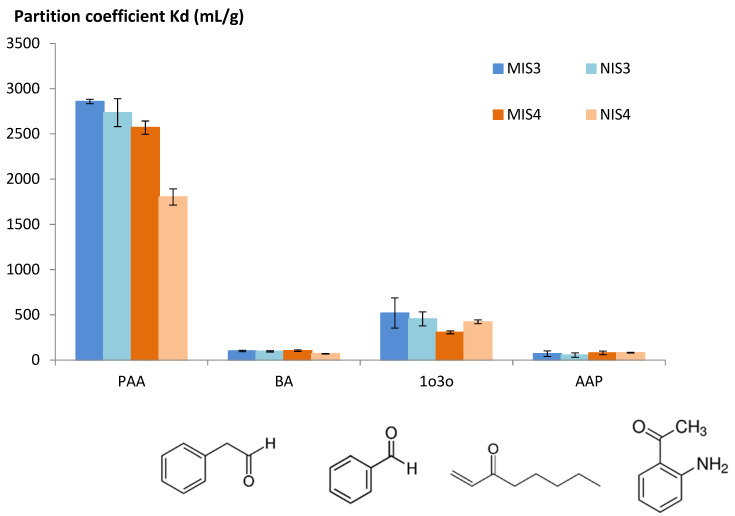
Partition coefficient in water/EtOH (90/10; *v*/*v*). Phenylacetaldehyde (PAA), Benzaldehyde (BA), 1-octen-3-one (1o3o) and 2-aminoacetophenone (AAP). Error bars represent standard deviation.

The selectivity study was conducted in hydro alcoholic medium in the presence of phenylacetaldehyde and three competing molecules: benzaldehyde (BA), 1-octene-3-one (1o3o) and 2′-aminoacetophenone (AAP). BA has a chemical structure very similar to PAA with one less carbon atom. 1o3o has a chemical structure very different from PAA: it is a linear structure without the aromatic ring. AAP represents and intermediate interfering compound. Selectivity results were presented as partition coefficients between the initial solution of PAA or the interferent and the polymer (Figure 2).

Both MIS3 and MIS4 gave high partition coefficients for PAA in comparison with competing molecules. They are then highly selective to PAA. No difference was observed between MIS3 and NIS3; this could be explained by the absence of a steric effect in the imprinting process. Only chemical complementarity affected the behavior of MIS3 and NIS3 since they were synthesized using the same functional monomer.

In the case of PAA, polymer 4 was the only one to show a difference between the printed and unprinted polymer. In this case, the steric effect and the chemical complementarity between the functional monomer and the template explain the MIS/NIS difference.

In conclusion and based on the previous results, MIS4 was the best candidate to be integrated in a microwave sensor to detect PAA in red wine: MIS4 presented a high sorption of PAA (37 mg⋅g^−1^), a satisfactory difference between MIS and the corresponding control NIS and a good selectivity for PAA. Morphologic characterizations were conducted on MIS4 and NIS4 before their deposition on the surface of a microwave antenna.

### 2.4. Characterization of MIS4 and NIS4

**Figure 3:**
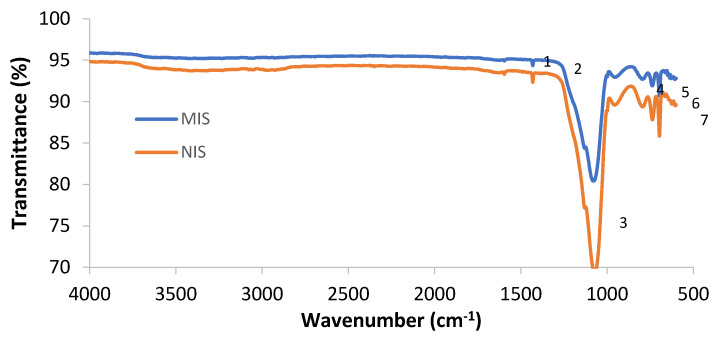
Infrared spectra of MIS4 and NIS4. Absorption assignements: (1) 1597 cm^−1^ (C=C) aromatic, (2) 1431 cm^−1^ (Si-phenyl), (3) 1075 cm^−1^ (Si-O-Si), (4) 950 cm^−1^ (Si-O^-^), (5) 790 cm^−1^ (Si-O-Si), (6) 738 cm^−1^ (Si-phenyl), (7) 698 cm^−1^ (Si-phenyl).

MIS4 and NIS4 were characterized by MID infrared spectroscopy to check their chemical structure. The corresponding infrared spectra are presented in Figure 3.

MIS4 and NIS4 have identic IR spectra. All the observed peaks are related to the absorption of the TEOS crosslinker and the PTMS monomer after the sol–gel hydrolysis and condensation. The MIS and NIS spectra are similar because the MIS was washed in order to release the template. The signature of the template cannot be observed. The FTIR analysis has the main objective to show that the polymerization occurred: the Si-O-Si bonds were formed, and the monomer was immobilized in the polymer. The condensed PTMS has several absorption bands: peaks at 1431, 738 and 698 cm^−1^ correspond to the Si-phenyl group and the one at 1597 cm^−1^ corresponds to the C=C aromatic group. The condensed TEOS has several absorption bands: at 1075 cm^−1^ corresponding to the Si-O-Si asymmetric stretching, at 950 cm^−1^ corresponding to Si–O^−^ stretching and at 790 cm^−1^ corresponding to the Si-O-Si symmetric stretching.

**Figure 4:**
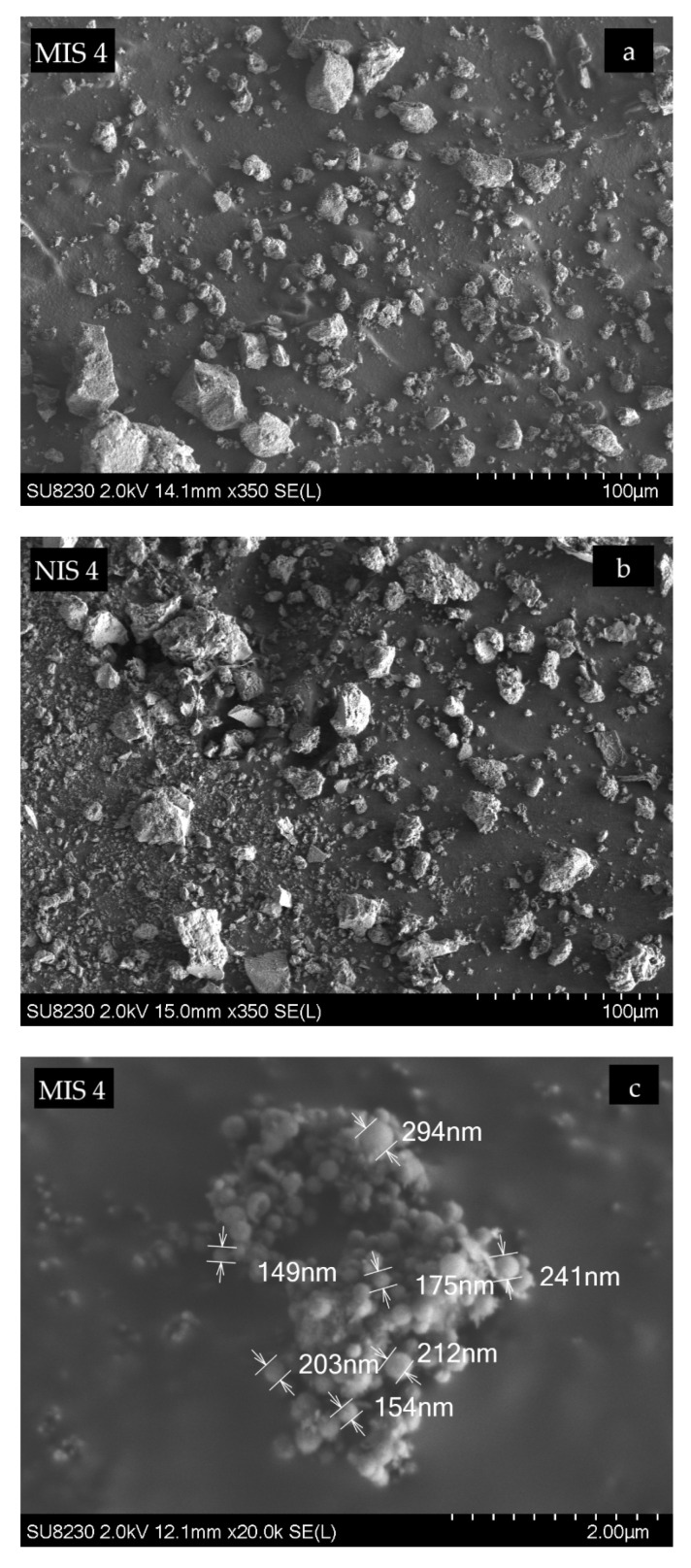

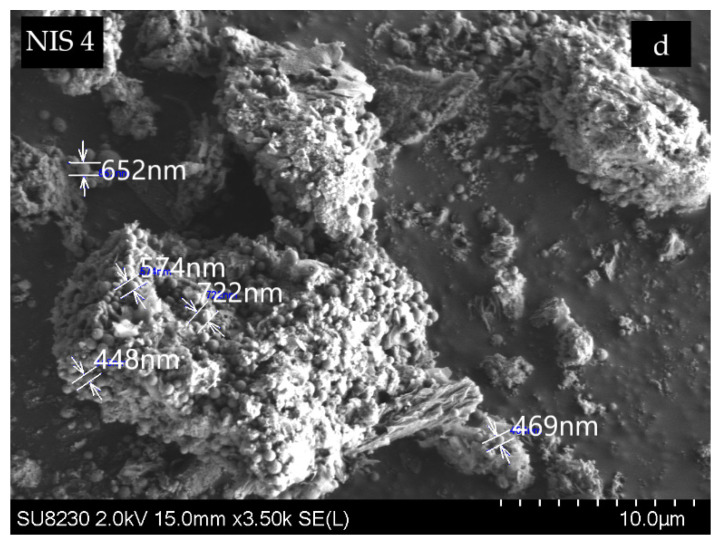
Scanning electron micrographs (SEM) for MIS4 and NIS4. (**a**) MIS4 at 350 magnifications. (**b**) NIS4 at 350 magnifications. (**c**) MIS4 at 20,000 magnifications. (**d**) NIS4 at 3 500 magnifications.

The morphology of MIS4 and NIS4 was assessed by SEM studies. Figure 4 shows the SEM micrographs of the MIS4 at two magnifications: 350 (a) and 20,000 (c) and a micrograph of NIS4 at 2 magnifications: 350 (b) and 3500 (d).

The MIS4 polymer seems to be an aggregate of regular and spherical particles. The aggregate size is less than 100 µm and the average particle size is about 200 nm. The NIS4 polymer seems to be an aggregate of irregular particles with a larger size compared with the MIS. The aggregate size is less than 300 µm and the average particle size is about 570 nm.

ATG results show that the percentage of grafted material was 37.6% for the MIS4 and 31% for the NIS4. It was calculated using the following formula:

(1) $$Grafted\;material\;\left(\%\right)=\frac{\left(m_{i}-m_{f}\right)}{m_{i}}\times 100$$

where *m_i_* and *m_f_* are the initial and final mass of the sample at 150 °C and 1000 °C, respectively.

### 2.5. Measurements Using the Microwave Sensor

**Figure 5:**
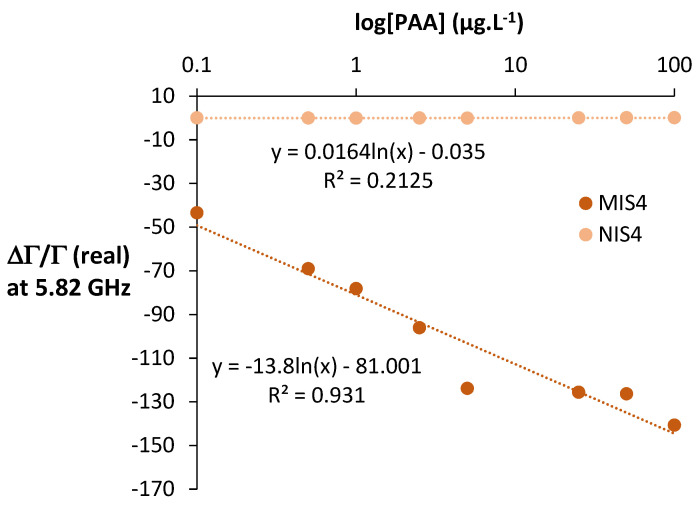
Effect of PAA concentration on the response of the MIS/NIS-microwave sensor in water/EtOH (90/10; *v*/*v*). Uncertainty on ∆Γ/Γ = 0.1%. The correlation coefficients for linear correlation are over the critical values of the Pearson’s correlation coefficient in a one-tailed test at the level of significance of 1%. 8 points, 6 degree of freedom, R critical value = 0.789 < R observed = 0.965.

After the deposition of the imprinted and the non-imprinted polymers (MIS4 and NIS4) on the surface of the microwave sensor, the microwave signal was measured at different concentrations of phenylacetaldehyde in hydro alcoholic medium (Figure 5).

The MIS-based sensor showed a linear response in hydro alcoholic medium for PAA concentrations between 0.1 and 100 µg⋅L^−1^. An important variation of the signal in function of the PAA concentration was observed: 97% between the two farthest points. The NIS-based sensor response was independent of PAA concentration and the corresponding signal remained at zero whatever the PAA concentration. This result shows that the molecular imprinting procedure was efficient. The lowest detected concentration in this case was 0.1 µg⋅L^−1^_._

**Figure 6:**
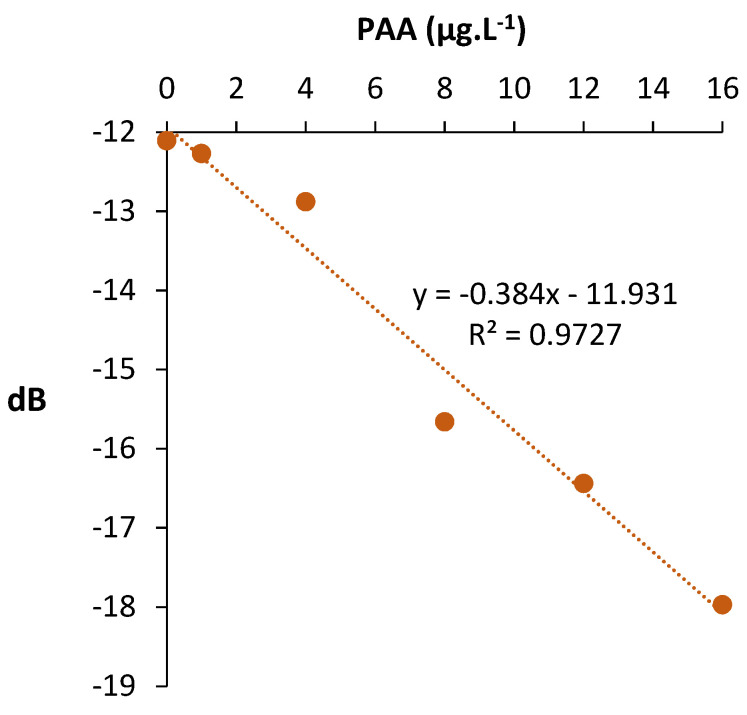
Effect of PAA concentration on the response of the MIS-microwave sensor in red wine at 2.4 GHz. Uncertainty on dB = 1%. The correlation coefficients for linear correlation are over the critical values of the Pearson’s correlation coefficient in a one-tailed test at the level of significance of 1%. 6 points, 4 degree of freedom, R critical value = 0.882 < R observed = 0.986.

Once the device was successfully applied in model wine, the PAA content of a Burgundy red wine was determined using a standard addition method. Results are shown in Figure 6.

The microwave signal (amplitude) was proportional to PAA concentration between 1 and 16 µg⋅L^−1^. Molecularly imprinted-based sensors were thus able to detect phenylacetaldehyde in red wine at the µg·L^−1^ level below the off-flavor threshold. The lowest detected concentration in red wine using the microwave sensor was 1 µg⋅L^−1^. This value is lower than the limit of detection of PAA determined by solid phase micro-extraction coupled to gas chromatography mass spectrometry (SPME-GCMS) [15].

To conclude, one of the main organoleptic defaults in wine is oxidation. Phenylacetaldehyde is a good chemical indicator of the oxidation level. Its aroma threshold varies between 1 and 25 µg⋅L^−1^. The classical method of phenylacetaldehyde assessment is gas chromatography coupled to mass spectrometry. GC-MS is time-consuming and expensive. In this work, a microwave sensor having as sensitive material a molecularly imprinted silica was developed as a fast and cheap method for the analysis of PAA. The developed microwave sensor was able to detect PAA in red wine at the µg·L^−1^ level.

## 3. Materials and Methods

### 3.1. Chemiclas

Phenylacetaldehyde (PAA 95%, CAS number 122-78-1), Benzaldehyde (BA ≥ 99%, CAS number 100-52-7), 1-octen-3-one (1o3o 96%, CAS number 4312-99-6), 2′-aminoacetophenone (AAP 98%, CAS number 551-93-9), (3-Aminopropyl)trimethoxysilane (APTMS 97%, CAS number 13822-56-5), [3-(Phenylamino)propyl]trimethoxysilane (PATMS 97%, CAS number 3068-76-6), phenyltrimethoxysilane (PTMS 97%, CAS number 2996-92-1), tetraethoxysilane (TEOS ≥ 99%, CAS number 78-10-4), ammonium hydroxide (NH_4_OH 28–30%, CAS Number 1336-21-6), hydrochloric acid (HCl 0.1 mol⋅L^−1^, Titripur, CAS number 7647-01-0) and ethanol (≥99.8%, CAS number 64-17-5) were purchased from Sigma Aldrich, St. Quentin Fallavier, France. S1813 photosensitive resin and MF319 developer were purchased from Chimie Tech Service, Antony, France. Polydimethylsiloxanes (PDMS) was obtained from FARNELL, Limonest, France. The water used in all experiments was deionized and obtained from an Elga Ionic system PURELAB Option. The model wine consisted of water/ethanol (90/10, *v*/*v*) solutions. Red wine from Burgundy was bought from a grocery store (Carrefour, Quetigny, France).

### 3.2. Molecularly Imprinted Silica Synthesis

MIS polymers were prepared at 40 °C in a thermostatic water bath under magnetic stirring. The template molecule was first solubilized in ethanol. Then, water was added, followed by the functional monomer and the crosslinker TEOS. Finally, NH_4_OH or HCl was introduced in order to initiate base or acid condensation. The reaction mixture was left under stirring for 20 h. The polymers were separated from the liquid phase by centrifugation at 10,000× *g* for 10 min at room temperature. In order to eliminate the template, polymers were washed several times with ethanol until it was no longer detectable by chromatography in washing solvents. After washing, polymers were dried for 6 h at 60 °C. In parallel, non-imprinted silica NIS were synthesized under the same synthesis conditions as those of MIS, but without PAA, the template molecule. NIS served as control polymers.

### 3.3. Batch Extraction Studies of PAA in Hydro Alcoholic Medium

The tested polymers were suspended in water/EtOH (90/10; *v*/*v*). A stock solution of water/ethanol with PAA was prepared at 400 mg·L^−1^. Three concentrations were studied: 20, 80 and 200 mg⋅L^−1^. Tests were conducted in 2 mL Eppendorf tubes in triplicate. In each tube, 1 mg of polymer (MIS or NIS) was contacted with PAA solutions at room temperature. The tubes were agitated in an orbital shaker for 2 h at 20 rpm, then centrifuged 10 min at 15,000× *g*. In order to determine the PAA concentration in the supernatant, 500 µL of the latter was mixed with 500 µL of 1-octanol internal standard solution and analysed by Gas Chromatography Mass Spectrometry (GC-MS). The amount of PAA sorbed to the polymer (mg/g), was calculated from the difference between the initial and the free PAA.

### 3.4. Batch Selectivity Studies in Hydro Alcoholic Medium

The selectivity studies were performed the same way as the batch extraction study. PAA was replaced by a mixture of competing molecules: phenylacetaldehyde (PAA), Benzaldehyde (BA), 1-octen-3-one (1o3o) and 2-aminoacetophenone (AAP). The initial concentration of each of these molecules was 400 mg⋅L^−1^. The partition coefficient (*Kp*) of each compound between the polymer and the hydro alcoholic medium was used to assess the selectivity. It was calculated using the following equation:

(2) $$Kp=\frac{C_{i}-C_{f}}{C_{f}}\times \frac{V}{m}$$

where *C_i_* is the initial concentration of the target compound, *C_f_* the concentration at equilibrium, *V* the solution volume and m the polymer mass. *C_i_* and *C_f_* were determined by GC-MS using internal standardization method. All experiments were carried out in triplicate.

### 3.5. Gas Chromatography Mass Spectrometry (GC-MS)

One µL of solutions prepared in paragraphs 3.3 and 3.4. was injected in split mode (split ratio15:1) using an auto-sampler on a 5973 gas chromatograph (Hewlett-Packard, Palo Alto, CA, USA) equipped with a fused-silica capillary column (30 m × 0.32 mm ID, 0.5 µm film thickness) coated with a DB-Wax stationary phase (J & W Scientific, Santa Clara, CA, USA). The injection temperature was 240 °C. Helium was used as the carrier gas at a flow rate of 1.4 mL⋅min^−1^ and the chromatographic temperature was programmed from 100 °C (initial time 2 min) to 220 °C at a rate of 10 °C⋅min^−1^, with a final isotherm of 5 min. Mass spectrometry was taken in the electron ionization mode at 70 eV and the scan range between 29 and 350 amu. The ion source was set at 230 °C and the transfer line at 250 °C. Compounds were identified by comparison with mass spectra libraries (WILLEY138 and NIST).

### 3.6. Characterisation of the Molecularly Imprinted and Non-Imprinted Sol–Gel Polymers

Fourier-transform infrared (FTIR) spectra were recorded on a Perkin Elmer spectrum 65 FT-IR spectrometer in the range 4000–600 cm^−1^ using attenuated total reflectance sampling. 64 scans with a resolution of 4 cm^−1^ were carried out for each polymer powder. For the surface morphological characterization, samples were suspended in ethanol; then, a drop was placed on a silicon grid and examined in a JEOL-7600 scanning electron microscope (SEM). The thermogravimetric analyses were carried out in a TA SDT Q600 instrument. The experiments were performed under air flow using a temperature increase of 20 °C per minute. Samples were heated from room temperature to 1000 °C.

### 3.7. Microwave Sensor Design and Measurement

**Figure 7:**
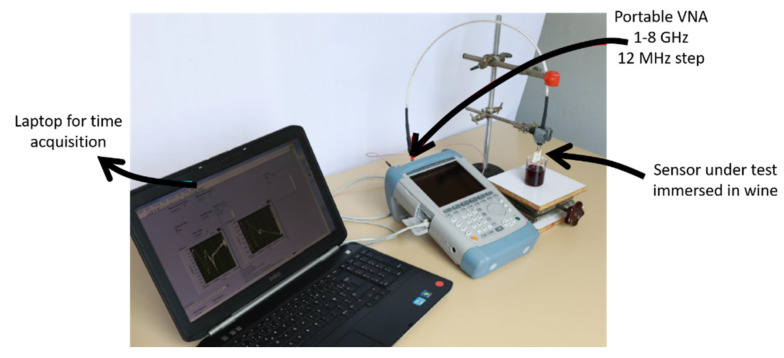
Experimental setup of the microwave measurement test bench.

The microwave transduction is based on the electromagnetic wave excitation in a sensitive material inside a propagative structure (coplanar sensor) in the range of microwave (between 1 and 8 GHz). The reflected and transmitted waves are determined using a Vector Network Analyser (VNA). The experimental setup (Figure 7) includes a portable VNA connected to a computer. The VNA and its cable (included the phase of calibration) are mechanically stable to avoid perturbations of the signal. The sensor was immersed in 100 mL of red wine alone (bank) or red wine supplemented with PAA. The concentration of PAA was varied using the standard addition method. The stock solution was prepared in water/EtOH (90/10; *v*/*v*) and was at 10 mg⋅L^−1^.

The microwave sensor consists of a microstrip transmission line with a trapezoidal spiral [16,17]. The proposed circuit was obtained by making the sensor on Rogers RT/duroid^®^ 6202 substrate (*ε_r_* = 2.94). Trapezoidal spiral was made of 7 copper strips with a space of 0.150 mm between the segments. The detailed dimensions and comparison with the full wave simulation (CST) are given in the previous work [16]. Except the spiral, the entire sensor is coated with polydimethylsiloxanes (PDMS). The MIS sensitive material was deposited following a simple Dr. Blade protocol.

The microwave sensor response at a specific frequency (*f*) was characterized by the reflection coefficient *Γ*(*f*) = *Re*(*f*) + *i*⋅*Im*(*f*). Re represents the real part of the coefficient and Im the imaginary part. The exploited signals were obtained from the relative variation of the reflection coefficient during the immersion in a sample, in comparison with the immersion in a reference blank solution (without target compounds). It was calculated from the following equation:

(3) $$\frac{∆\Gamma }{\Gamma }=\frac{\Gamma \left(sample\right)-\Gamma \left(blank\right)}{\Gamma \left(blank\right)}$$

Transformation and logarithmic scaling were implemented on *Γ*(*f*) coefficient giving: $A=20\log\left(\sqrt{Re^{2}+Im^{2}}\right)$ where A represents the amplitude in dB.

## Figures and Tables

**Table 1:** Experimental design for the synthesis of the five MIS/NIS.

Reagent/Polymer Name	MIS1	MIS2	MIS3	MIS4	MIS5
FM ^1^	PATMS	APTMS	PATMS	PTMS	APTMS
C ^2^	TEOS	TEOS	TEOS	TEOS	TEOS
I ^3^	NH_4_OH	NH_4_OH	NH_4_OH	NH_4_OH	HCl
S ^4^	W/EtOH ^5^ 50/50	W/EtOH 90/10	W/EtOH 90/10	W/EtOH 90/10	W/EtOH 90/10
Polymerisation	no	yes	yes	Yes	yes

^1^ FM = functional monomer, ^2^ C = Crosslinker, ^3^ I = Initiator, ^4^ S = Solvent, ^5^ W/EtOH = Water/Ethanol. PATMS = Propylaniline trimethoxysilane, APTMS = Aminopropyl trimethoxysilane, PTMS = Phenyl trimethoxysilane, TEOS = Tetraethoxysilane.

## Data Availability

Not applicable.

## Sample Availability

Samples are available from the authors.

## References

[1] Bueno M., Culleré L., Cacho J., Ferreira V. (2010). Chemical and Sensory Characterization of Oxidative Behavior in Different Wines. Food Res. Int..

[2] Wang H., Ramnani P., Pham T., Villarreal C.C., Yu X., Liu G., Mulchandani A. (2019). Gas Biosensor Arrays Based on Single-Stranded DNA-Functionalized Single-Walled Carbon Nanotubes for the Detection of Volatile Organic Compound Biomarkers Released by Huanglongbing Disease-Infected Citrus Trees. Sensors.

[3] Lu Y., Li H., Zhuang S., Zhang D., Zhang Q., Zhou J., Dong S., Liu Q., Wang P. (2014). Olfactory Biosensor Using Odorant-Binding Proteins from Honeybee: Ligands of Floral Odors and Pheromones Detection by Electrochemical Impedance. Sens. Actuators B Chem..

[4] Wen H., Wang H., Hai J., He S., Chen F., Wang B. (2019). Photochemical Synthesis of Porous CuFeSe_2_/Au Heterostructured Nanospheres as SERS Sensor for Ultrasensitive Detection of Lung Cancer Cells and Their Biomarkers. ACS Sustain. Chem. Eng..

[5] Wang H., Ramnani P., Pham T., Villarreal C.C., Yu X., Liu G., Mulchandani A. (2020). Asymptomatic Diagnosis of Huanglongbing Disease Using Metalloporphyrin Functionalized Single-Walled Carbon Nanotubes Sensor Arrays. Front. Chem..

[6] Svenson J., Nicholls I.A. (2001). On the Thermal and Chemical Stability of Molecularly Imprinted Polymers. Anal. Chim. Acta.

[7] Lafarge C., Bitar M., El Hosry L., Cayot P., Bou-Maroun E. (2020). Comparison of Molecularly Imprinted Polymers (MIP) and Sol–Gel Molecularly Imprinted Silica (MIS) for Fungicide in a Hydro Alcoholic Solution. Mater. Today Commun..

[8] Yang C.-L., Chang T.-C., Chen Y.-Y. Microwave Sensors Applying for Traditional Chinese Medicine Pulse Diagnosis. Proceedings of the 2017 International Workshop on Electromagnetics: Applications and Student Innovation Competition.

[9] Zhang K., Amineh R.K., Dong Z., Nadler D. (2019). Microwave Sensing of Water Quality. IEEE Access.

[10] Zarifi M.H., Deif S., Abdolrazzaghi M., Chen B., Ramsawak D., Amyotte M., Vahabisani N., Hashisho Z., Chen W., Daneshmand M. (2018). A Microwave Ring Resonator Sensor for Early Detection of Breaches in Pipeline Coatings. IEEE Trans. Ind. Electron..

[11] Trabelsi S., Nelson S.O. (2016). Microwave Sensing of Quality Attributes of Agricultural and Food Products. IEEE Instrum. Meas. Mag..

[12] Rossignol J., Dujourdy L., Stuerga D., Cayot P., Gougeon R.D., Bou-Maroun E. (2020). A First Tentative for Simultaneous Detection of Fungicides in Model and Real Wines by Microwave Sensor Coupled to Molecularly Imprinted Sol-Gel Polymers. Sensors.

[13] Bou-Maroun E., Rossignol J., De Fonseca B., Lafarge C., Gougeon R.D., Stuerga D., Cayot P. (2017). Feasibility of a Microwave Liquid Sensor Based on Molecularly Imprinted Sol-Gel Polymer for the Detection of Iprodione Fungicide. Sens. Actuators B Chem..

[14] Chiang C.-L., Ma C.-C.M., Wu D.-L., Kuan H.-C. (2003). Preparation, Characterization, and Properties of Novolac-Type Phenolic/SiO_2_ Hybrid Organic–Inorganic Nanocomposite Materials by Sol–Gel Method. J. Polym. Sci. Part A Polym. Chem..

[15] Jeong H.-S., Chung H., Song S.-H., Kim C.-I., Lee J.-G., Kim Y.-S. (2015). Validation and Determination of the Contents of Acetaldehyde and Formaldehyde in Foods. Toxicol. Res..

[16] Bailly G., Harrabi A., Rossignol J., Michel M., Stuerga D., Pribetich P. (2017). Microstrip Spiral Resonator For Microwave-Based Gas Sensing. IEEE Sens. Lett..

[17] Hallil H., Dejous C., Hage-Ali S., Elmazria O., Rossignol J., Stuerga D., Talbi A., Mazzamurro A., Joubert P.-Y., Lefeuvre E. (2021). Passive Resonant Sensors: Trends and Future Prospects. IEEE Sens. J..

